# Receptor–Receptor Interactions and Glial Cell Functions with a Special Focus on G Protein-Coupled Receptors

**DOI:** 10.3390/ijms22168656

**Published:** 2021-08-12

**Authors:** Diego Guidolin, Cinzia Tortorella, Manuela Marcoli, Chiara Cervetto, Guido Maura, Luigi F. Agnati

**Affiliations:** 1 Department of Neuroscience, Section of Anatomy, University of Padova, 35121 Padova, Italy; cinzia.tortorella@unipd.it; 2 Department of Pharmacy, Center of Excellence for Biomedical Research, University of Genova, 16126 Genova, Italy; marcoli@difar.unige.it (M.M.); cervetto@difar.unige.it (C.C.); maura@difar.unige.it (G.M.); 3 Department of Biomedical Sciences, University of Modena and Reggio Emilia, 41125 Modena, Italy; luigi.agnati@gmail.com

**Keywords:** glial cells, receptor–receptor interactions, oligomerization, allostery, GPCR

## Abstract

The discovery that receptors from all families can establish allosteric receptor–receptor interactions and variably associate to form receptor complexes operating as integrative input units endowed with a high functional and structural plasticity has expanded our understanding of intercellular communication. Regarding the nervous system, most research in the field has focused on neuronal populations and has led to the identification of many receptor complexes representing an important mechanism to fine-tune synaptic efficiency. Receptor–receptor interactions, however, also modulate glia–neuron and glia–glia intercellular communication, with significant consequences on synaptic activity and brain network plasticity. The research on this topic is probably still at the beginning and, here, available evidence will be reviewed and discussed. It may also be of potential interest from a pharmacological standpoint, opening the possibility to explore, inter alia, glia-based neuroprotective therapeutic strategies.

## 1. Introduction

More than 4% of the human genome encodes cell receptors [1], which are presently organized into different families (see [2]) including intracellular receptors, matrix receptors, ligand- and voltage-gated ion channels, enzyme-linked receptors and G protein-coupled receptors (GPCRs). In humans, GPCRs represent the largest family, made up of about 800 members further classified into five major groups (classes A, B, C, frizzled and adhesion) based on sequence homology and functional similarity [3]. From a structural standpoint, GPCR monomers are characterized by seven α-helixes piercing the plasma membrane, linked by extra- and intra-cellular loops [4]. It is well known that GPCR monomers can recognize and decode a variety of signals [5,6,7] and are also endowed with an intrinsic plasticity, as GPCR activation can lead to different transduction patterns, such as G protein and/or arrestin pathways [8,9].

In the early 1980s, however, in vitro and in vivo experiments [10,11,12] provided indirect evidence that GPCRs may also establish structural receptor–receptor interactions (RRI), leading to the formation at the cell membrane of multimeric receptor complexes (see [13,14,15] for reviews) that operate as integrative input units [16]. In the years that followed, direct evidence for the existence of this structural organization was provided by several groups [17,18,19,20,21,22,23,24,25,26,27], and the amount of data supporting the existence of GPCR oligomers further increased when biophysical techniques capable of detecting the spatial proximity of protein molecules became available [28,29].

These findings demonstrated that GPCRs can signal both as monomers and as part of receptor complexes and indicated that oligomeric organization represents a quite common feature in the different receptor families, with the ion channel receptors (where multimerization is needed) lying at one end of the spectrum and GPCRs at the other [30]. Receptor channels, indeed, are constitutively multimeric [31], the majority of nuclear hormone receptors operate as homo- or hetero-dimers [32] and, with few exceptions [33], receptor tyrosine kinases need dimerization for their activation [34]. Thus, as pointed out by Changeux and Christopoulos in a detailed review [35], oligomerization emerges as an efficient mechanism for tuning the functionality of receptor proteins, including those able to signal as monomers, such as GPCRs. In this respect, recently reported evidence for receptor complexes involving protomers from different families [30,36] is also of significant interest.

RRI at the cell membrane have expanded our understanding of intercellular communication and they appeared to play a major role in the physiology and pathology of many districts of the body (see [30] for a review). Examples include the regulation of vascular homeostasis through the angiotensin II AT_1_ receptor and its heterodimers [37], the chemoreceptor function of the carotid body [38] and the endocrine system, where a growing number of reports suggested receptor oligomerization as a significant aspect of endocrine regulation [39]. The possibility of pharmacological strategies targeting receptor heteromers has also been proposed in oncology [40]. However, the largest body of available data concerns the central nervous system (CNS). The formation of receptor complexes, indeed, is considered of key importance in neurophysiology (see [41,42,43,44] for more specific reviews), since the integration of input signals already at the level of the plasma membrane significantly helps to tune synaptic efficiency. Furthermore, increasing evidence indicates receptor complexes as potential targets for the treatment of serious diseases of the CNS [45,46,47].

In this context, glial cells, the non-action potential generating cells in the CNS, received less attention. More recently, however, the increased evidence that glial cells are not merely a support to neuronal life, but are actively involved in neuronal development, function and synaptic plasticity [48], generated an intense research interest focused on the mechanisms of glia–neural communication with significant new findings on the role played by receptor–receptor interactions in this process. Thus, after a brief recapitulation of the basic aspects concerning the structural biology of receptor complexes and their signaling, the available data on the role RRI play in the intercellular communication involving glial cells will be the focus of the present review article.

## 2. Structural Biology and Signaling of Receptor Complexes

It is well known that receptors can interact in a functional sense by sharing signaling patterns or by mechanisms of transactivation, even without coming into physical contact with each other [49]. The term RRI, on the contrary, indicates a type of interaction requiring direct physical contact between the receptors involved, leading to the formation of receptor complexes at the cell membrane. In this respect, a more detailed definition was provided in 2010 by a specific international consensus workshop [50]: “Receptor-receptor interactions: when the binding of a ligand to the orthosteric or allosteric sites of one receptor causes, via direct allosteric interactions, a change in the ligand recognition, decoding and trafficking processes of another receptor”. On this basis, it is also possible to provide an operational definition, in which the term RRI is translated into a set of experimental procedures leading to unambiguous numerical descriptions of the phenomenon [13]. In this respect, it is possible to maintain that two receptors are involved in an RRI process when the binding of one receptor causes detectable changes in the biochemical characteristics of the partner and the two receptor molecules are located in close proximity (<10 nm). In the last few decades, several biophysical techniques have been developed to detect the spatial proximity of protein molecules (see [15,28,29,45] for more details). They include energy transfer-based methods, bimolecular luminescence or fluorescence complementation, total internal reflection fluorescence microscopy, fluorescence correlation spectroscopy, coimmunoprecipitation, assays based on bivalent ligands and in situ proximity ligation assays.

### 2.1. RRI as Allosteric Interactions

Allostery (see [35,51,52,53] for extensive reviews) is a mode of communication between distant sites in a protein, in which the energy associated with dynamic or conformational changes at one site can be transferred (traveling along specific pathways within the protein structure) to other sites, changing their conformational or dynamic features accordingly. Since allostery involves changes in protein conformation, a protein with a rigid structure is less predisposed to be allosterically modulated than a protein characterized by plastic segments that do not fold into a stable secondary structure, as those endowed with intrinsic disorder [54]. In this respect, intrinsically disordered regions have been identified in all families of cell receptors and mechanisms of structural change between order to disorder (or vice versa) likely underlie their activation [30].

Structural plasticity, however, is important not only to allow intra-receptor interactions and conformational fluctuations, but also to enable the formation of receptor complexes and their dynamics. When protomers, indeed, establish direct RRI leading to a quaternary structure, energy perturbations occurring at some site of one protomer can propagate over the interface between receptors into the nearby protomers, changing their conformational and functional properties, thus allowing the cooperative behavior of the complex [55].

**Figure 1:**
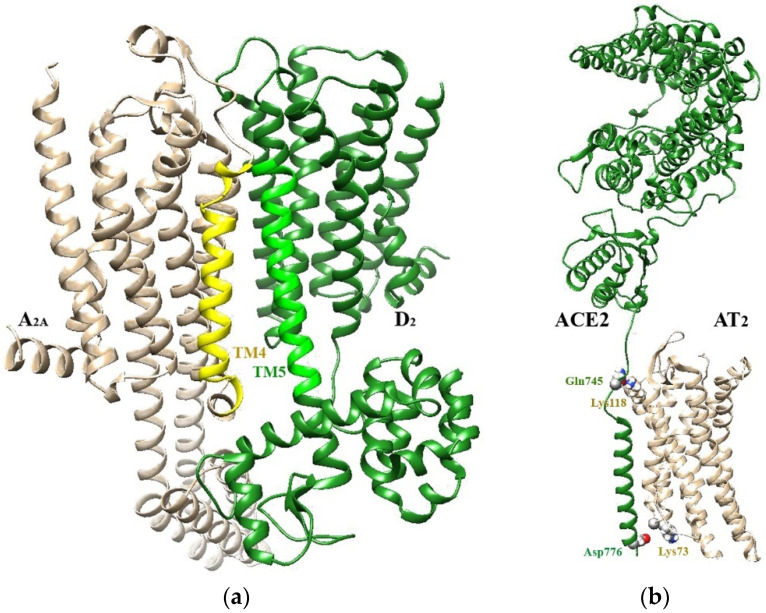
Models of heterodimers as estimated by bioinformatics methods: (**a**) A_2A_–D_2_ receptor complex according to [64]. The transmembrane domains TM4 and TM5 involved in the heterodimerization interface are emphasized; (**b**) Heterodimer between ACE2 and angiotensin AT_2_ receptors as predicted by docking and molecular dynamics methods [65]. Residues predicted to be involved in H-bonds between monomers are also indicated.

The identification of the residues that specifically interact to form the interface is therefore of significant interest in current research on receptor oligomerization, since they influence the overall architecture the receptor complex can assume. In this respect, several bioinformatics methods have been devised to predict the available interfaces (see [15,56,57,58]) They can, in principle, be categorized into two broad classes according to the type of input data used to perform the analysis. The first class of methods involves the analysis of the primary structure of the proteins under scrutiny to explore biophysical features of their amino acid sequence, allowing the identification of sites potentially involved in an interaction. Procedures aimed at finding intrinsically disordered domains [59,60] and strategies based on the identification of functional residues conserved during evolution (see [56]) belong to this group. Although quite powerful in identifying residues that are essential for RRI, these tools, however, often provide a number of “false positives” [58]. A more direct approach is provided by the second class of methods requiring the analysis of the tertiary structure of the proteins under study to identify possible surfaces for interaction. In this context, a presently often used strategy to estimate the structure of receptor complexes involves the application of docking methods [61], followed by a refinement of the estimated quaternary structure by energy minimization [62] or molecular dynamics (MD) protocols [63]. Some recently proposed structures [64,65] are provided in Figure 1. Furthermore, several improvements in experimental procedures have provided a range of methods to test the suggestions coming from bioinformatics. They include advanced crystallization techniques [66], atomic force microscopy [55] and novel super-resolution imaging approaches [67]. An interesting finding emerging from computational and experimental studies on oligomerization interfaces is the presence at the interface of specific motifs that appear of particular importance for allosteric interactions. Examples include the electrostatic interactions between intra-cellular domains demonstrated by Woods and colleagues [68] in GPCRs and the so-called SmallxxxSmall motifs that are part of the dimer interface in receptor tyrosine kinases [69] and in some GPCRs [70] as well. SmallxxxSmall motifs are characterized by the presence of small amino acids (such as Ala, Gly, Ser, Thr) in i, i + 4 positions and determine the interaction between trans-membrane helices. A further aspect of substantial interest concerns the kinetics of complex formation and its dependence on the interaction energy. GPCRs provide an example [15,71]. To exist as a stable dimer with a half-life comparable to that of even short-lived GPCRs (2–20 h), an interaction energy of at least −60 kJ/mol is required. This condition is often fulfilled by class C GPCRs, explaining why they usually appear as stable dimers, but not by class A GPCRs, which often form transient receptor dimers leading to a dynamic equilibrium condition at the cell membrane with the constant formation and dissociation of new receptor complexes.

### 2.2. Signaling from Receptor Complexes

The establishment of these supramolecular assemblies is considered of particular importance because it allows the emergence of integrative functions performed by a receptor complex as a whole [15]. In fact, owing to allosteric RRI, a configuration change of a given protomer will change the probability of changing the configuration for the adjacent receptors in the complex and the effect will propagate throughout the cluster, leading to complex collective behavior and to an integrated regulation of multiple effectors. These concepts have been well illustrated by mathematical models of cooperativity in receptor assemblies [58], based on discrete dynamics [72] or on thermodynamics-based approaches [73]. In the former case, receptors are supposed to assume a limited number of configurations (e.g., only two: “active” or “inactive”) changing in the time according to a “switching rule” based on the pattern of interactions each receptor establishes with the partners in the complex. In the latter case, the transition is stochastic and depends on the estimated energy of each protomer in the complex (see [58] for more details). Mathematical models indicated that receptor complexes can be described as possessing “emergent properties”, i.e., biochemical and functional features that cannot be fully anticipated on the basis of the characteristics of the single receptor partners [74].

Thus, when RRI take place at the membrane, the actual signaling outcomes of receptor complexes depend on several factors, including the composition of the complex and its topological organization, the traffic of the receptor complex, the effects of ligands on the formation and the stability of the assembly, and the possible crosstalk with other signaling pathways [15,30,75]. Together, these factors may strongly influence the chain of events linking ligand recognition to signal transduction from the single protomers. Some basic signaling consequences that the allosteric interactions may induce should be considered [15,30].

In a variety of receptor complexes, the modulation of the binding sites has been reported as a consequence of allosteric RRI. Examples include the heterodimer between adenosine A_2A_ and dopamine D_2_ GPCRs [45], where reciprocal antagonism occurs, and the human insulin RTK [76], a glycoprotein existing in two dimeric isoforms that exhibit significant differences in affinity for insulin-like growth factors. Changes in the decoding of signals reaching protomers represent a second mechanism induced by allosteric RRI. This aspect seems of particular importance in GPCRs, as illustrated by the heterodimer formed by dopamine D_1_ and histamine H_3_ receptors [77], in which the D_1_ receptor changes its coupling from the G_s_ to the G_i_ protein, or by the switch from G protein to β-arrestin signaling [78] documented after κ-μ and κ-δ opioid receptor oligomerization. A final relevant aspect of receptor complex formation is the possibility that novel specific allosteric sites suitable for the binding of some modulators could appear in the quaternary structure resulting from the assemblage of protomers [59]. Thus, ligands specific to the receptor complex as such may also exist.

## 3. Receptor–Receptor Interactions in Glial Cells

Glial cell processes are uniquely positioned to receive signals from neurons and to signal back to them in order to provide structural support to neurons and synapses. Emerging evidence, however, demonstrated that bidirectional signaling also leads to the involvement of glial cells in the active regulation of neuronal and synaptic function [79,80]. This dense crosstalk between cell populations of the CNS is mediated by the release of neuronal and glial transmitters acting on glial and neuronal receptors, respectively. Thus, in the last few years, interest in the potential role the mechanism of RRI may play in regulating glial cells’ behavior has increased, and this research effort allowed the identification of an increasing number of receptor complexes operating in glial cells. Available evidence is summarized in Table 1 and a more detailed discussion will be provided in the sections that follow.

### 3.1. Astrocytes

It is well known that a number of astrocyte homeostatic functions support neuronal activity. They include water regulation in the neuronal microenvironment [98] and metabolic support to neurons [99]. These homeostatic and maintenance roles certainly impact on synaptic efficiency. Increasing evidence, however, indicates a more direct involvement of astrocytes in the regulation of neuronal excitability and action potential propagation, as demonstrated by studies on excitatory synapses leading to the proposal of the concept of “tripartite synapse” [100]. According to this view, the relationship between astrocytes and neurons is a bidirectional one, with neural activity influencing astrocytic activation, which in turn modulates the activity of neurons [80]. To monitor the extracellular environment, indeed, astrocytes express specific receptors and channels (see [101]). Notably, astrocytes can express many neurotransmitter receptors also expressed by neurons, allowing them to respond to a variety of neuronal signals [102]. Available evidence indicates that single astrocytes integrate incoming information through the elevation of intracellular Ca^2+^ in the cells [103], and it is well established that astrocytes can propagate this information over large distances by communicating with each other through calcium waves [79]. Such calcium dynamics, mediated by gap junctions, is considered an important mechanism leading to the release of gliotransmitters (d-serine, ATP, glutamate) from astrocytes and to a direct regulation of ongoing neural activity [104]. As indicated by a number of experimental studies (reviewed in [80]), this intercellular crosstalk significantly influences both short- and long-term synaptic plasticity and, as a consequence, higher CNS functions such as learning and memory [80]. The exact mechanisms and spatiotemporal scales of astrocytic gliotransmission and related processes are still under careful investigation [105]. In this context, however, many available data indicate that RRI may play a significant role.

**Figure 2:**
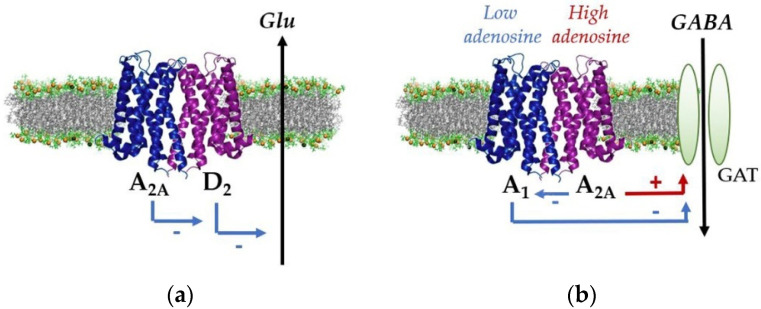
Schematic view of signaling processes driven by receptor complexes in astrocytes: (**a**) Modulation of the release of glutamate by the A_2A_–D_2_ receptor complex [81]. Dopamine D_2_ receptor activation inhibits the release of glutamate, while activation of adenosine A_2A_ receptors abolishes the effect of D_2_ activation; (**b**) Control of GABA uptake through GABA transporters (GAT) by the adenosine A_1_–A_2A_ receptor complex [87] (see text).

The first example is provided by adenosine A_2A_ and dopamine D_2_ receptors (see Figure 2a). Both receptor types were found co-expressed on the same astrocyte processes [81] where they form receptor heteromers, as quite recently demonstrated by using biochemical and biophysical techniques, such as co-immunoprecipitation and proximity ligation assay [82]. From the functional standpoint, A_2A_–D_2_ RRI were tested by measuring in vitro the release of the gliotransmitter glutamate following the administration of the D_2_ receptor agonist quinpirole and the A_2A_ receptor agonist CGS21680 [81]. The activation of D_2_ receptors inhibited glutamate release, while the activation of A_2A_ receptors, per se ineffective, abolished the release inhibition induced by D_2_ activation. Interestingly, the administration of the synthetic peptide VLRRRRKRVN, which can interfere with the D_2_ receptor domain involved in electrostatic interactions critical to receptor heteromerization [68], eliminated the A_2A_-mediated inhibition of the response to D_2_ receptor activation, indicating that receptor complexes were responsible for the observed effect. Consistently, the D_2_-mediated inhibition of glutamate release by astrocytes was also reduced by intracellular homocysteine [83], a known allosteric modulator of the A_2A_–D_2_ receptor complex [59].

Depression or enhancement of synaptic plasticity may also result from cannabinoid receptor-mediated astrocyte activation and the release of gliotransmitter ATP/adenosine, as suggested by studies on the basolateral amygdala [106]. In this respect, the identification by proximity ligation assay of cannabinoid CB_2_ and GPR55 receptor complexes in the astrocytes of the dorsolateral prefrontal cortex of the human brain [84] is of potential interest. From the functional point of view, the results revealed an association between the expression levels of this heteromer and mood disorders, but no data are still available on the signaling features specific to this receptor complex.

Of interest in the study of neurological disorders with cognitive decline is the recent demonstration by proximity ligation assay of receptor complexes involving fibroblast growth factor receptor 1 (FGFR1) and serotonin 5HT_1A_ receptor in hippocampal astrocytes [85]. The FGFR1–5HT_1A_ heteroreceptor complex may allow astroglial modulation of the hippocampal neurons’ gamma oscillations, a pattern of electrical activity (30–80 Hz) playing an important role in cognitive processes, such as memory storage and recall.

Inhibition is a fundamental operational mechanism in the brain, mainly governed by GABAergic interneurons. In the neocortex, key populations of these cells are parvalbumin (PV)- and somatostatin (SST)-expressing interneurons, regulating the spike-timing and the synaptic plasticity of pyramidal neurons [107]. By using optogenetics and two-photon functional imaging, it has been shown that these two interneuron classes differentially signal to astrocytes inducing weak and robust GABA_B_ receptor-mediated Ca^2+^ elevations, respectively, allowing astrocytes to produce additional modulatory actions in local brain circuits [86]. A finding of interest for the present discussion is that the higher sensitivity exhibited by the astrocytic GABA_B_ receptor in response to SST-interneurons depends on the co-released peptide somatostatin. Post-embedding electron microscopy experiments revealed that cortical astrocytes express both GABA_B_ and somatostatin SSTR4 receptors with similar densities at perisynaptic astrocytic processes, where GABA_B_–SSTR4 couples (<50 nm) were detected, opening the possibility that the observed effect could be based on allosteric RRI between the two receptors [86].

Astrocytes modulate synaptic transmission not only by releasing gliotransmitters, but also by uptaking neurotransmitters to fine-tune the balance between excitation and inhibition [108]. A level of regulation of GABA uptake is under the control of extracellular adenosine and occurs via the modulation of GABA transporters by the adenosine A_1_ and A_2A_ receptors (Figure 2b). In this respect, in vitro studies [87] demonstrated by co-immunoprecipitation and BRET assay that in astrocytes these receptors can be organized as A_1_–A_2A_ receptor complexes, coupled to two distinct G proteins, regulating GABA transport in an opposite way, with the A_1_ protomer mediating inhibition and the A_2A_ protomer mediating facilitation of GABA transport. At low levels, adenosine preferentially binds the A_1_ protomer, activating G_i/0_ protein, while at high concentrations adenosine activates the A_2A_ protomer, inhibiting the partner and, through G_s_ protein, leading to an enhancement of GABA uptake. The receptor complex, therefore, was suggested to operate as a dual amplifier to control ambient GABA levels at synapses [87].

During development, astrocytes are of key importance to drive the formation of synapses, and many neuronal populations (e.g., spinal motor neurons, GABAergic and glycinergic neurons) show a limited ability to form functional synapses in the absence of astrocytes [79]. In the complex network of intercellular crosstalk involved in developmental processes, RRI may represent a significant regulatory mechanism. The receptor complex between serotonin 5HT_1A_ and dopamine D_2_ receptors likely provides an example. About 35% of the total D_2_ receptor binding activity in the cortex may be associated with astrocytes [109], and the 5HT_1A_ receptor is also well expressed in these cells, where its stimulation regulates the growth of serotonergic neurons [110]. By using proximity ligation assay and fluorescence energy transfer, the two receptors were found to co-localize in astrocytes and form 5HT_1A_–D_2_ heteromers [88]. Moreover, treatment with a multireceptor antagonist (such as risperidone) facilitated the heteromerization, leading to an increased ERK1/2 activity, an important event which may regulate synaptic plasticity, development and repair [111]. A mechanism of potential interest in the early phases of postnatal plasticity [112] may also be a functional partnership between metabotropic glutamate receptors mGluR3 and mGluR5 observed in astrocytes characterized by a reactive-type phenotype, where endogenous activation of mGluR3 is required for maximal mGluR5 signaling [89]. However, no evidence demonstrating the formation of mGluR3–mGluR5 receptor complexes has been provided so far.

Instead, a demonstrated heteromeric association in astrocytes involves adenosine A_1_ and purinergic P2Y_1_ receptors [90]: these receptors co-localize on astroglial membranes in rat hippocampus where they organize into receptor complexes, as indicated by co-immunoprecipitation methods. From the functional standpoint, the results indicated that within the complex P2Y_1_ receptor activation induces A_1_ receptor desensitization. Thus, it has been suggested that this heteromer could play an important role in the astrocytic modulation of glutamatergic neurotransmission during pathological conditions (e.g., tissue damage and inflammation), when large amounts of adenosine and purines are released [90].

### 3.2. Microglia

Microglia are the resident immune system of the CNS, representing 5–10% of the brain cell population. They are characterized by two key functional features (see [80,113]): immune defense and maintenance of tissue homeostasis. Thus, to perform their functions microglial cells express many types of receptors [80].

A quite large set of receptors allows them to detect molecular patterns associated with tissue damage, to modulate the release of cytokines and to facilitate phagocytosis. In this context, of potential interest for the present discussion are P2X (ligand-gated cationic channels) purinergic receptors. As a matter of fact, P2X_4_ and P2X_7_ are the dominant forms of P2X receptors expressed in microglia [114]. Although still a matter of debate (see [115]), the possible occurrence of P2X_4_–P2X_7_ heteromers has been reported in these cells [91], probably allowing a more sophisticated regulation of cytokine production and early inflammatory gene expression [91,114].

**Figure 3:**
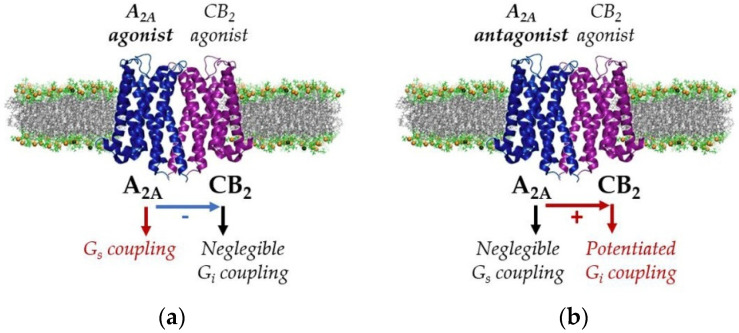
Schematic view of signaling processes driven by the A_2A_–CB_2_ receptor complex in microglia [94]. cAMP determination assays are consistent with G_s_ coupling to A_2A_ and with G_i_ coupling to CB_2_. (**a**) Upon simultaneous treatment using the two agonists, the G_i_ coupling was negligible, indicating that A_2A_ activation blocks CB_2_-mediated signaling; (**b**) The administration of antagonists of the A_2A_ receptor (e.g., SCH58621) blocked the effect of the A_2A_ agonist and potentiated CB_2_ receptor signaling in the A_2A_–CB_2_ heteromer context.

Microglia, however, can also be neuroprotective, since at least two phenotypes (M1 or proinflammatory and M2 or neuroprotective) may arise from the activation of resting (or M0) cells [116]. In this respect, evidence exists suggesting an important potential of microglial cannabinoid receptors in the regulation of M1/M2 polarization [117]. Studies in this field allowed the identification of a number of heteroreceptor complexes involving cannabinoid receptors in microglia. The first example is the receptor complex CB_1_–CB_2_ between the two types of cannabinoid receptors, being particularly well expressed in activated microglia [92,93], where the activation of one receptor blunts the response of the partner in the heteromer, leading to a wide spectrum of effects when reached by endocannabinoids or by synthetic molecules acting on cannabinoid receptors [118]. Associations of the cannabinoid CB_2_ receptor with the adenosine A_2A_ receptor [94] or with the orphan receptor GPR18 [95] to form heteromers were also demonstrated in activated microglia. In the A_2A_–CB_2_ receptor complex (see Figure 3), the blockade of the A_2A_ receptor leads to increased CB_2_ signaling [94], while bidirectional cross-antagonism was observed in the GPR18–CB_2_ heteromer [95]. In view of the fact that the activation of the CB_2_ receptor is generally considered as anti-inflammatory [119], both the receptor complexes are considered of interest from a pharmacological standpoint, since the use of antagonists targeting A_2A_ or GPR18 receptors could be useful in the microglia-mediated protection of neuronal death in neurodegenerative diseases [94,95].

### 3.3. Oligodendrocytes and Schwann Cells

Oligodendrocytes represent the myelinating cells in the CNS and derive from oligodendrocyte progenitor cells (OPCs) generated in restricted regions of the developing neural tube, such as the ventral spinal cord, the floor of the third ventricle, and the medial ganglionic eminence (see [113]). OPCs then migrate to appropriate axon-rich regions to become mature oligodendrocytes and provide myelination. OPCs persist into adulthood and are capable of proliferating and subsequently differentiating into myelinating oligodendrocytes throughout life. The proliferation and differentiation of OPCs are modulated by growth factors, as well as by communication between OPCs and neurons, with OPCs being the only glial cells receiving direct synaptic input [120].

In this respect, of particular interest for the present discussion is the pivotal role played by GABAergic signaling [96]. Interestingly, indeed, the cells of the oligodendrocyte lineage express the metabotropic GABA receptor GABA_B_, which is a well-known heterodimer [121], formed by two GPCR subunits (GABA_B1_ and GABA_B2_). However, the ratio of GABA_B1_ to GABA_B2_ changes with the differentiation of OPCs into oligodendrocytes, suggesting that B1 and B2 subunits could also form homodimers or interact with other membrane elements [96]. From a functional standpoint, GABA_B_ signaling has been found to promote cell migration and myelination (at least in vitro) [96].

In the peripheral nervous system, Schwann cells are the myelinating cells and neuregulins represent an example of axonally derived ligands interacting with cognate receptors in Schwann cells to regulate their development and proliferation [122]. The receptors involved are erb2 and erb3, which become tyrosine phosphorylated and form erb2–erb3 heterodimers upon ligand binding [97].

## 4. Concluding Remarks and Perspectives

Intercellular communication represents a key feature of living organisms, and in the nervous system it determines virtually all aspects of its function. The main mechanism of communication in biological tissues involves the interaction of chemicals and/or energy forms released from a source with specific receptors expressed by the target cells. In the last few decades, the emerging evidence that receptors from all families can establish allosteric RRI and variably associate to form receptor complexes [30] indicated RRI as a basic mechanism modulating and tuning intercellular communication [15]. In a receptor complex, indeed, the configuration of each single receptor is shaped by a network of electrostatic interactions (hydrogen bonds and Van der Waals forces) defined by the presence of receptor partners, thereby enabling the complex to operate as an integrative input unit [16,55].

As far as the nervous system is concerned, the research effort to identify and characterize RRI was mainly focused on neurons and clearly indicated this mechanism as a relevant factor contributing to set and tune the synaptic strength [13,14] and, consequently, impacting on the emerging field of “connectomics” [41]. This line of research allowed the identification of a high number of RRI, most presently stored in specific databases (see [123,124]). Intercellular communication in the nervous system, however, is not limited to neurons. More recently, emerging evidence pointed to the significant involvement of glia–glia and glia–neuron communication in modulating and shaping synaptic activity and plasticity [48,80], leading to a deeper understanding of how glial cells contribute to information processing within the neural circuitry [101]. The study of the role played by RRI in this context is probably still at the beginning, and the number of receptor complexes identified so far in glial cells is still limited. Although further studies are needed to expand this knowledge, the available data seem to indicate that the bidirectional glia–neuron signaling at synapses may find in RRI a significant regulatory mechanism giving high flexibility to intercellular communication at this level [125].

Several additional lines of future research, however, can be identified. In the CNS, indeed, chemical transmitters are released in two distinct transmission modes: wiring transmission and volume transmission (see [41,126] for reviews). Wiring transmission (WT) is intercellular communication mediated via physically defined connection structures. Synapses and related glial processes represent the typical example. Volume transmission (VT) occurs by the release and diffusion of chemical signals in the extracellular space defined by the intricate morphological organization of neurons, glial cells and extracellular matrix [123]. It is primarily mediated by simple diffusion, but also by pressure waves due to the arterial pulses, thermal gradients and local electric fields [127]. VT signals can be released from any type of brain cells and can be sensed by a relatively large number of cells, including microglia [128] and astrocytes [129,130,131]. VT mainly employs the same set of signals (transmitters, peptides, ions, gases) as WT (see [41] for a summary table). An important finding, however, was that non-synaptic receptors are usually characterized by high affinity for the signal [132]. Thus, an interesting topic for future research in the field could be a differential analysis of the glial and neural receptor complexes involved in the two forms of intercellular communication to assess possible differences in their signaling features. The analysis of such an issue could likely require a more detailed description of the cellular localization of the receptor complexes. As summarized before (see Table 1), this aspect has been addressed only to a limited extent, with the majority of available studies being aimed only at demonstrating the presence of receptor complexes in the cells. In some cell populations, however, this issue could be of significant physiological importance, as indicated by the increasing number of studies revealing the existence of functional microdomains in astrocytes (see [133] for a recent review). The term “microdomains” describes Ca^2+^ events that are restricted to small portions of individual astrocyte territories and can either remain restricted locally or eventually propagate to the main processes and to the soma of the cells. The characterization of the panel of receptors and receptor complexes associated with these sub-cellular functional domains could, therefore, represent a key step to increase our understanding of the astrocyte role in brain function. This issue, however, poses some methodological challenges. Important techniques currently used to demonstrate receptor complexes, such as proximity ligation assays, also provide morphological information on their location. However, the obtainable resolution at light microscopy may be a limitation and the development of more suitable imaging techniques would be beneficial. In this respect, procedures based on 3D super-resolution microscopy [134], electron microscopy [86], and atomic force microscopy [55] supported by specific image analysis methods [55] have been suggested and may represent topics for further methodological development.

Receptor complexes are also of interest from a pharmacological standpoint, and their pharmacology certainly represents a significant line of future research. RRI, indeed, may provide new opportunities to optimize existing pharmacological treatments or to develop completely new pharmacological strategies. In this respect, the use of agonists/antagonists of single protomers in the receptor complexes has been, to some extent, successfully explored [45]. However, the search for receptor heteromers’ selective compounds would be of key importance to fully exploit their properties. At least three approaches could be followed to achieve this goal. The first is based on the fact that, due to a different pattern of allosteric RRI, the conformational state of a given protomer may change according to the type of complex in which it is involved [135]. Thus, the pharmacology of some agonists/antagonists of a given protomer in terms of affinity and efficacy may show substantial differences among various types of receptor complexes. A second approach to identify receptor complex selective compounds is based on the possibility that, when the complex forms, the quaternary structure could display novel specific allosteric sites suitable for the binding of some modulators. The abovementioned effect of homocysteine on astrocytic A_2A_–D_2_ receptor complexes [83] provides an example. The use of bivalent ligands constitutes a third possible approach for targeting receptor heteromers (see [136] for a review). A bivalent ligand consists of two pharmacophoric entities linked by an appropriate spacer. In this way, it should be possible to target GPCR heteromers by adequate, potent, and receptor-selective pharmacophores. The work of Portoghese and collaborators on opioid receptor complexes (see [137]) provided a proof of principle. In this research effort, bioinformatics can be of help and MD simulations appear of particular importance in the field, since they allow the analysis of the conformational dynamics of receptors and receptor complexes in a realistic model of their biological environment, including the lipid bilayer and the extra- and intra-cellular water spaces [138]. MD methods, however, are in general computationally demanding and require specific software and expertise. On-line resources, however, are becoming available to facilitate MD data acquisition and analysis, and some of them are specifically designed to support studies on receptor proteins [139].

When applied to glial receptor complexes, these pharmacological research lines can represent a topic of particular interest from a therapeutical standpoint. Indeed, as suggested by some of the available studies discussed here, they open the possibility to explore novel, glia-mediated strategies to address neurodegenerative [93,94] and functional [84,85,140] CNS disorders.

## Figures and Tables

**Table 1:** Receptor complexes identified in glial cell populations.

Glial Cell Population	Receptor Complex	Cellular Localization	Reference
Astrocytes	A2A–D2	Striatal astrocyte processes	[81,82,83]
	CB2–GPR55	Plasma membrane	[84]
	FGFR1–5HT1A	Plasma membrane	[85]
	GABAB–SSTR4 (probable)	Cortical astrocyte processes	[86]
	A1–A2A	Plasma membrane	[87]
	5HT1A–D2	Mainly cell soma	[88]
	mGluR3–mGluR5 (putative)	Not reported	[89]
	A1–P2Y1	Plasma membrane	[90]
Microglia	P2X4–P2X7	Plasma membrane	[91]
	CB1–CB2	Plasma membrane	[92,93]
	A2A–CB2	Plasma membrane	[94]
	GPR18–CB2	Plasma membrane	[95]
Myelinating cells	GABAB1–GABAB2	OPC–neuron contacts	[96]
	erb2–erb3	Not reported	[97]

## Data Availability

Data sharing not applicable, since no new data were recorded or analyzed in this study.
